# Case count metric for comparative analysis of entity resolution results

**DOI:** 10.3389/fdata.2026.1736939

**Published:** 2026-06-11

**Authors:** John R. Talburt, Muzakkiruddin Ahmed Mohammed, Mert Can Cakmak, Onais Khan Mohammed, Mahboob Khan Mohammed, Khizer Syed, Leon Claassens

**Affiliations:** 1 Center for Advanced Research in Entity Resolution and Information Quality (ERIQ), University of Arkansas at Little Rock, Little Rock, AR, United States; 2 Pilog Group, Centurion, South Africa

**Keywords:** clustering evaluation, comparative analysis, data quality, entity resolution, evaluation metrics, record linkage

## Abstract

**Introduction:**

Entity resolution (ER) systems often produce different clustering outcomes when applied to the same dataset, especially when parameters, algorithms, or system configurations change. However, in many real-world settings, the true linking structure is unknown, making traditional accuracy-based evaluation difficult.

**Methods:**

This paper presents the Case Count Metric System (CCMS), a process and software system for comparing two cluster ER outcomes without requiring a truth set. CCMS classifies how each cluster from the first ER process is transformed by the second process into four mutually exclusive cases: unchanged, merged, partitioned, or overlapping.

**Results:**

CCMS produces aggregate case counts, singleton summaries, and per-cluster transformation details to support diagnostic analysis. Example applications using synthetic demographic data and an industrial materials dataset show that CCMS can identify how clustering outcomes change under parameter adjustments and alternative ER systems.

**Discussion:**

CCMS provides a practical and interpretable method for comparing ER clustering results when labeled ground truth is unavailable. By distinguishing between over-linking, under-linking, and more complex cluster reorganizations, CCMS offers more actionable insight than single-value similarity measures and supports both research analysis and operational ER evaluation.

## 1 Introduction

Entity Resolution (ER) is crucial for effective data management, particularly when dealing with large and complex datasets. ER is usually defined as the process of determining if two information system references (sometimes called “mentions” or “observations”) to real world objects are referring to the same, or to different, objects (Binette and Steorts, 2022) In the case that two references are to the same object, the references are said to be equivalent (Talburt, 2011).

ER is usually implemented in one of two forms, cluster ER or binary ER (Binette and Steorts, 2022). The most basic and oldest ER process is binary ER (Fellegi and Sunter, 1969) initially developed to reconcile census of a given population taken at different times. In binary ER, the input comprises two distinct datasets with the goal of identifying and linking equivalent references between the two datasets. Often the assumption is that there are no equivalent references within each dataset and as a result, a reference in the first dataset can link to at most one reference in the second dataset resulting in one-to-one binary ER. If this condition is relaxed, the linking can be one-to-many between the datasets.

However, the focus of this paper is on cluster ER. In cluster ER, in the input is a single dataset and the objective is to partition the dataset into non-intersecting subsets (clusters) where each cluster represents the entirety of references in the dataset referencing a particular entity. A formal mathematical definition of the entity resolution of a given set of references was developed at the Stanford InfoLab (Benjelloun et al., 2009). In cluster ER, all the references in the same cluster are equivalent, and references in different clusters are not equivalent. In terms of a graph where the nodes are references and links are edges, a cluster is a maximally connected component of the graph.

While binary ER relies entirely on direct linking of references, i.e., matching references that meet a specific level of similarity, cluster ER includes two additional indirect linking processes. The first indirect linking process is transitive closure. If one accepts the unique reference assumption for the input dataset (Talburt and Zhou, 2015) then reference equivalence is a true equivalence relation in the mathematical sense, i.e., it is a binary set relation that is idempotent, symmetric, and transitive (Rotman, 2010). This allows non-matching references to be indirectly linked as equivalent through a chain of direct matching links. For example, if Reference A and Reference B are considered equivalent because A matches B, and if Reference B and C are considered equivalent because B matches C, then by transitive closure it follows that A and C are equivalent even if A and C do not match. The addition of indirect linking sometimes leads to confusion in the evaluation of the binary ER results versus cluster ER results. In binary ER, only direct matches are evaluated as either true or false positive links. However, in cluster ER, all pairs of references in the same cluster (both directly and indirectly linked) are considered linked pairs and subject to evaluation as either true positive or false positive links (Ye and Talburt, 2018).

The second indirect linking process in cluster ER is by patterns. A common indirect linking pattern is the household move pattern often observed in demographic data where members of the same household are observed residing at two or more addresses (Fu et al., 2014). For example, the four-way pattern that person named John Doe is found residing at both Oak Street and Elm Street, and that another person named Mary Doe is also found residing at the same two addresses provides evidence (an increased probability) that these could be the same persons. While the two John Doe references and the two Mary Doe references might not rise to level of a matching pair, they still might be indirectly linked based on this household move pattern. Especially if there is additional evidence such as age similarity, low frequency names, more than two household members, or additional addresses for the same household members. The implementation of the proposed method and experimental scripts are publicly available on GitHub.

## 2 Problem statement

Master data management (MDM) is a widely used data curation process employed by most large organizations to facilitate accurate data integration at scale. Cluster entity resolution (ER) is a foundational process in all MDM systems. In practice, cluster ER systems often generate multiple plausible clustering outcomes from the same underlying dataset. These differences arise because cluster ER processes typically involve a number of configurable decisions, heuristics, and parameters that directly influence how references are grouped into entities.

Most cluster ER systems operate as multi-stage pipelines that include blocking, pairwise comparison, similarity scoring, and cluster formation through transitive closure or other consolidation rules. Variations in blocking strategies determine which record pairs are considered for comparison, while adjustments to similarity thresholds and feature weighting influence which candidate pairs are linked. In addition, indirect linking mechanisms such as transitive closure and pattern-based linking can cause local linking decisions to propagate through clusters, resulting in merges, partitions, or more complex reorganizations. As a result, even modest parameter changes or algorithmic interventions can lead to materially different clustering outcomes.

From an academic perspective, cluster ER performance is commonly evaluated using metrics such as Precision, Recall, F-measure, or weighted variants of these measures (Christen et al., 2023). While these metrics can be computed to compare different ER outcomes, their interpretation as measures of accuracy relies on the availability of a truth set that allows record pairs to be judged as true positives, false positives, true negatives, or false negatives. When a truth set is available, it is straightforward to compare ER outcomes and assess relative accuracy. However, for most real-world applications, the true linking structure for any sizeable collection of references is unknown. Although stratified sampling techniques can be used to estimate Precision and Recall for large datasets (Pullen, 2017; Penning, 2016), these approaches are labor-intensive and time-consuming.

Beyond Precision, Recall, and F-measure, additional methods exist for evaluating cluster ER outcomes. Confusion matrix analyses that summarize false positive and false negative links are recommended by Herzog et al. (2007) as a means of understanding error types and comparing system accuracy. However, these methods likewise depend on the availability of a linking truth set.

When assessing ER system performance under varying decision criteria, Receiver Operating Characteristic (ROC) curves and the Area Under the Curve (AUC) are also commonly used. These tools are particularly useful for tuning ER systems to satisfy operational objectives, such as minimizing false negatives in fraud detection or increasing precision in targeted marketing applications (Hand and Christen, 2018). As with other accuracy-oriented metrics, their interpretation depends on labeled ground truth.

In operational and commercial ER deployments, investigators therefore often explore multiple clustering configurations, either by tuning parameters within a single system or by comparing outputs from different ER systems, without access to ground truth. This creates a need for evaluation methods that can systematically characterize and interpret differences between clustering outcomes without relying on labeled data, motivating the development of the Case Count Metric System (CCMS) introduced in this paper.

## 3 Related work

A variety of approaches have been proposed for evaluating entity resolution (ER) outcomes, particularly in settings where labeled ground truth is limited or unavailable. This section reviews the most relevant lines of work, organized by evaluation strategy, and positions the Case Count Metric System (CCMS) relative to existing methods.

### 3.1 Background: ER evaluation context

Entity resolution has been studied under various names—duplicate detection, record linkage, deduplication, co-reference resolution—across multiple research communities since the foundational work of Newcombe et al. (1959) and Fellegi and Sunter (1969). Comprehensive surveys by Elmagarmid et al. (2007), Getoor and Machanavajjhala (2012), and Christophides et al. (2021) document the breadth of ER methods and the corresponding diversity of evaluation strategies. As ER systems have grown in sophistication, incorporating blocking (Papadakis et al., 2020), probabilistic models (Murray, 2016), Bayesian methods (Steorts et al., 2016), deep learning architectures (Mudgal et al., 2018; Li et al., 2020), and, more recently, multi-agent retrieval-augmented generation frameworks for household-level entity resolution (Althaf et al., 2025), the number of plausible clustering configurations that practitioners must compare and evaluate has increased substantially.

Evaluation of ER systems has traditionally relied on one of three strategies: benchmarking on standardized datasets, testing on synthetic or simulated data, or case studies in operational settings. Benchmarking provides controlled comparisons using datasets such as the Cora citation dataset, the Restaurant dataset, and the Febrl synthetic datasets (K"opcke et al., 2010; Christen, 2012), but benchmark performance does not guarantee effectiveness on operational data. Simulated data—including synthetically generated demographic records (Talburt et al., 2009) and census-style datasets (Haddock et al., 2024)—can alleviate the truth set problem but depend critically on how well simulated error distributions match real-world patterns. Case studies (Winkler, 2006) illuminate practical deployment challenges but are constrained by confidentiality and are rarely accompanied by complete ground truth. All three strategies face limitations when the goal is to compare ER outcomes on operational data where the true linking structure is unknown.

### 3.2 Pairwise evaluation metrics

The most basic evaluation approach computes pairwise Precision, Recall, and F-measure by classifying every pair of records that share a cluster as either a true positive or a false positive link, with pairs separated across clusters evaluated as true negatives or false negatives (Christen, 2012; Herzog et al., 2007). While straightforward, pairwise metrics have well-documented limitations. The number of record pairs grows quadratically with cluster size, so pairwise metrics can be dominated by large clusters and may not accurately reflect entity-level performance. A system that places all records into a single cluster achieves perfect Recall but very low Precision, while a system that assigns each record to its own cluster achieves perfect Precision but very low Recall. Confusion matrix analyses that summarize false positive and false negative links, as recommended by Herzog et al. (2007), provide additional diagnostic information but likewise depend on the availability of a linking truth set. Receiver Operating Characteristic (ROC) curves and the Area Under the Curve (AUC) are useful for tuning ER systems to satisfy operational objectives (Hand and Christen, 2018), but their interpretation also requires labeled ground truth.

### 3.3 Cluster-level evaluation metrics

To address the limitations of pairwise metrics, several cluster-level evaluation metrics have been proposed, primarily in the co-reference resolution literature but widely adopted in ER evaluation.

#### 3.3.1 MUC

The MUC metric, introduced by Vilain et al. (1995), evaluates co-reference by counting the minimum number of links needed to partition or merge system-produced clusters to match the reference clustering. MUC Recall measures how many links must be added to the system output to reconstruct the reference clusters, while MUC Precision measures how many links must be removed. While intuitive, the MUC metric has a well-known deficiency: it does not penalize systems that place all records into a single cluster, because such a system requires no additional links to cover all reference entities (Amig'o et al., 2009). This bias toward large clusters limits MUC's utility for evaluating over-linking errors.

#### 3.3.2 B-Cubed

The B-Cubed metric, proposed by Bagga and Baldwin (1998), addresses MUC's limitation by computing per-record precision and recall. For each record, B-Cubed Precision measures the proportion of records in the same system cluster that also share a reference cluster, and B-Cubed Recall measures the proportion of records in the same reference cluster that also share a system cluster. The per-record scores are then averaged across all records. B-Cubed has been widely adopted in both co-reference resolution and ER evaluation because it provides a balanced assessment of over-linking and under-linking errors at the individual record level and does not exhibit the large-cluster bias of MUC.

#### 3.3.3 CEAF

The CEAF metric (Luo, 2005) takes a fundamentally different approach by finding an optimal one-to-one alignment between system-produced clusters and reference clusters using the Kuhn-Munkres algorithm, and then computing similarity based on this alignment. CEAF exists in two variants: entity-based CEAF (CEAF-e), which measures the overlap between aligned cluster pairs, and mention-based CEAF (CEAF-m), which counts the number of correctly aligned mentions. CEAF avoids the double-counting issues inherent in pairwise metrics and provides an entity-centric evaluation perspective. However, the one-to-one alignment constraint means that CEAF cannot account for situations where a single reference cluster is split across multiple system clusters (or vice versa) without penalizing the unaligned portions.

#### 3.3.4 BLANC

The BLANC metric (Recasens and Hovy, 2011) extends the Rand Index to coreference evaluation by separately computing precision and recall over coreferent (within-entity) and non-coreferent (between-entity) record pairs. BLANC averages the F-measures of these two pair types, providing a balanced evaluation that accounts for both linking and non-linking decisions. Unlike B-Cubed, which operates at the record level, BLANC operates at the pair level but avoids the large-cluster dominance problem of naive pairwise metrics by treating coreferent and non-coreferent pairs symmetrically. BLANC is particularly useful in settings where the number of entities is large relative to the number of records, as it gives appropriate credit for correctly separating non-coreferent pairs.

#### 3.3.5 LEA

The LEA (Link-based Entity-Aware) metric, proposed by Moosavi and Strube (2016), was motivated by the observation that existing metrics can disagree substantially on the relative ranking of ER systems and that none fully satisfies all desirable evaluation properties simultaneously. LEA computes entity-level scores by weighting each entity's contribution by its size and measuring the proportion of correct links within each entity. Specifically, LEA Recall for a reference entity is the proportion of within-entity record pairs that are correctly placed in the same system cluster, weighted by entity size. LEA Precision is defined analogously from the system cluster perspective. By incorporating both entity-level awareness and link-level evaluation, LEA addresses several known deficiencies of earlier metrics. LEA is particularly relevant to the present work because it shares the motivation of providing entity-level diagnostic insight, although LEA still requires a truth set for computation.

### 3.4 Comparative analysis and the metric selection problem

Menestrina et al. (2010) conducted a systematic comparison of several ER evaluation metrics, including pairwise F-measure, cluster-level metrics, and variations thereof. Their analysis demonstrated that different metrics can rank the same set of ER systems in different orders, highlighting the importance of metric choice and the challenge of selecting a single “best” evaluation measure.

This problem was formalized by Amig'o et al. (2009), who proposed a set of formal constraints that any clustering evaluation metric should satisfy, including constraints related to cluster homogeneity, completeness, rag bag (avoiding trivial solutions), and cluster size sensitivity. Their key result is that *no single existing metric satisfies all desirable constraints simultaneously*. B-Cubed satisfies the most constraints among the metrics they examined, but it still exhibits limitations in certain edge cases. This impossibility-type result has important implications for ER evaluation: it means that relying on any single metric—whether pairwise, cluster-level, or information-theoretic—provides an incomplete picture of ER quality. This finding directly motivates the development of complementary evaluation tools such as CCMS, which provide a different type of diagnostic information rather than attempting to replace existing metrics.

Hassanzadeh et al. (2009) proposed a framework specifically designed for evaluating clustering algorithms in the duplicate detection setting. Their framework introduces a set of quality measures that assess clustering outputs along multiple dimensions, including cluster purity, completeness, and the number of clusters relative to the ground truth. While their framework still requires ground truth for evaluation, it shares with CCMS the recognition that a single aggregate score is insufficient for understanding clustering behavior and that multi-dimensional assessment is needed.

Recent work has revisited and systematized ER evaluation frameworks, emphasizing the gap between idealized evaluation settings and realistic deployment scenarios (Wang et al., 2022), as well as proposing entity-centric evaluation frameworks that move beyond purely pairwise metrics (Binette et al., 2024).

### 3.5 Clustering comparison methods without ground truth

A related body of work in statistics and machine learning addresses the problem of comparing two partitions (clusterings) of the same dataset without requiring either partition to be designated as ground truth. These methods are directly relevant to the setting addressed by CCMS, in which two ER outcomes must be compared in the absence of a reference labeling.

The Rand Index (Rand, 1971) is the most basic measure for comparing two partitions. It computes the proportion of record pairs on which the two partitions agree, i.e., pairs that are either placed in the same cluster by both partitions or in different clusters by both partitions. The Adjusted Rand Index (ARI) (Hubert and Arabie, 1985) corrects the Rand Index for chance agreement, providing a normalized score where 0.0 indicates agreement no better than random and 1.0 indicates perfect agreement.

The Variation of Information (VI), introduced by (Meilă 2007), provides an information-theoretic distance between two clusterings based on the conditional entropies of each clustering given the other. VI satisfies the metric axioms (it is non-negative, symmetric, and satisfies the triangle inequality), making it a true distance measure on the space of partitions. Normalized Mutual Information (NMI) (Vinh et al., 2010) provides a related information-theoretic similarity measure that is normalized to the [0, 1] interval.

While these methods are well-suited for quantifying the overall degree of agreement or disagreement between two clusterings, they share a fundamental limitation for diagnostic purposes: each produces a *single scalar value* that summarizes how much two clusterings differ without revealing *how* or *why* they differ. A Rand Index of 0.85, for example, indicates substantial but imperfect agreement but does not indicate whether the disagreements are due to clusters being merged, split, or structurally reorganized. This distinction is precisely what is needed for diagnosing ER system behavior, tuning parameters, and prioritizing manual review.

### 3.6 The TWI metric

One prior effort that specifically addresses the comparison of ER clustering outcomes without ground truth is the TWI metric (Talburt et al., 2008). The TWI quantifies the overall degree of difference between two clustering results *A* and *B* over the same dataset and is defined as


$$TWI=\frac{\sqrt{\left|A|\cdot |B\right|}}{\left|V\right|}$$

where |*A*| and |*B*| denote the number of clusters in outcomes *A* and *B*, respectively, and |*V*| is the number of non-empty intersections between clusters in *A* and *B*. The TWI is normalized to the [0, 1] interval, where 1.0 indicates identical clusterings.

Like the Rand Index, ARI, VI, and NMI, the TWI produces a single scalar summary of clustering divergence. While useful as an overall indicator, it does not reveal whether observed differences are attributable to cluster merges, partitions, or more complex structural reorganizations.

### 3.7 Positioning of CCMS

The Case Count Metric System (CCMS) introduced in this paper is designed to be complementary to the metrics and methods reviewed above rather than a replacement for any of them. When a truth set is available, metrics such as B-Cubed, MUC, CEAF, BLANC, LEA, and pairwise F-measure provide precise measures of ER accuracy and should be used. When the goal is to obtain a single summary of how similar two clusterings are, measures such as the Rand Index, ARI, VI, NMI, or TWI are appropriate.

CCMS addresses a different and complementary need: given two ER clustering outcomes over the same dataset, CCMS characterizes *how* the clusters differ by decomposing the transformation from one clustering to the other into four mutually exclusive and exhaustive categories: Unchanged, Merged, Partitioned, and Overlapping. This structural decomposition provides diagnostic insight that scalar similarity measures cannot offer. In particular, CCMS enables practitioners to determine whether observed differences between ER outcomes are driven primarily by over-linking (merged cases), under-linking (partitioned cases), or more complex reorganizations (overlapping cases), and to drill down into specific cluster transformations for manual inspection.

The motivation for CCMS is closely aligned with the findings of Amig'o et al. (2009) and Hassanzadeh et al. (2009): since no single metric captures all dimensions of clustering quality, complementary tools that provide qualitatively different types of diagnostic information are valuable. Where existing metrics answer the question “how accurate is this ER outcome?” or “how similar are these two clusterings?,” CCMS answers the question “in what structural ways do these two clusterings differ, and which specific clusters are affected?”

Importantly, CCMS does not require a truth set and can be applied in any setting where two clustering outcomes are available for comparison. This makes it particularly suitable for operational ER deployments where ground truth is unavailable and investigators must compare parameter configurations, algorithmic variants, or entirely different ER systems.

Table 1 summarizes the key characteristics of the evaluation approaches reviewed in this section and positions CCMS relative to them.

**Table 1:** Comparison of ER evaluation approaches.

Method	Requires truth set	Output type	Explains how	Supports drill-down
Pairwise P/R/F	Yes	Scalar(s)	No	No
B-Cubed	Yes	Scalar(s)	No	No
MUC	Yes	Scalar(s)	No	No
CEAF	Yes	Scalar(s)	No	No
BLANC	Yes	Scalar(s)	No	No
LEA	Yes	Scalar(s)	No	No
Rand index/ARI	No	Scalar	No	No
VI/NMI	No	Scalar	No	No
TWI	No	Scalar	No	No
**CCMS**	**No**	**4 counts + details**	**Yes**	**Yes**

“Explains How” indicates whether the method characterizes the nature of clustering differences (merges, partitions, and overlaps). “Supports Drill-Down” indicates whether individual cluster transformations can be inspected. Bold values highlight the results obtained using the proposed CCMS metric system.

## 4 Methodology

Recognizing the limitations inherent in existing metrics and methods, this paper proposes a novel method, the Cluster Count Metric System (CCMS), specifically designed to provide a more granular analysis of the dynamics of cluster ER changes without recourse to a truth set.

### 4.1 The cluster count metric system

Let *ER*_1_ represent the set of clusters generated by the first (baseline) ER process acting on a given set of entity references *R*. Let *ER*_2_ represent the set of clusters generated by the second ER process acting on *R*. *ER*_1_ and *ER*_2_ are the inputs to CCMS, and the output produced by CCMS is a series of counts.

The first set of counts produced by CCMS provides an overall profile of *R*, *ER*_1_, and *ER*_2_. These include:

- *RC*= Count of references in *R*.
- *CC*_1_= Count of clusters generated by *ER*_1_.
- *SC*_1_= Count of singleton clusters generated by *ER*_1_ (singleton clusters are clusters containing only one reference).
- *CC*_2_= Count of clusters generated by *ER*_2_.
- *SC*_2_= Count of singleton clusters generated by *ER*_2_.

However, the primary CCMS output is a second set of four counts that describe how each cluster formed by *ER*_1_ is transformed by *ER*_2_. CCMS recognizes four distinct, mutually exclusive transformations of a cluster in *ER*_1_ to one or more clusters in *ER*_2_. For a given cluster *A*_*ER*1_, these counts are described as follows:

**Unchanged count (UC):** A count of cases where the *ER*_1_ cluster is identical to an *ER*_2_ cluster. In other words, through their linking decisions, both *ER*_1_ and *ER*_2_ have formed the same cluster of references.


$$A=B,\;\text{where }B\in ER_{2}$$

**Merged count (MC):** A count of cases where the entire *ER*_1_ cluster is a proper subset of an *ER*_2_ cluster. The *ER*_1_ cluster becomes part of (merges into) a larger *ER*_2_ cluster. In this case, all the references in the *ER*_1_ cluster were also linked by the *ER*_2_ process, but the *ER*_2_ process made additional links to these references that were not made by the *ER*_1_ process.


$$A\subset B\;\text{and}\;A\neq B,\;\text{where }B\in ER_{2}$$

**Partitioned count (PC):** A count of cases where the *ER*_1_ cluster is decomposed into multiple *ER*_2_ clusters. In this case, the *ER*_2_ process partitioned the *ER*_1_ cluster into smaller clusters without adding any new references. Each of the *ER*_2_ clusters having a non-empty intersection with the *ER*_1_ cluster is a proper subset of the *ER*_1_ cluster.


$$A=\overset{n}{\underset{i=1}{⋃}}B_{i},\;\text{where }B_{i}\in ER_{2}\;\text{and}\;n>1$$

**Overlapping Count (OC):** A count of the cases where the *ER*_1_ cluster has a non-empty intersection with two or more *ER*_2_ clusters and at least one intersecting *ER*_2_ is not a subset of the *ER*_1_ cluster. In other words, the *ER*_1_ cluster is a proper subset of the union of the *ER*_2_ clusters having a non-empty intersection with the *ER*_1_ cluster. Unlike the partitioned case, this indicates an overlap where the *ER*_1_ cluster is divided, but the resulting *ER*_2_ clusters include more references than the original, suggesting a more complex reorganization.


$$A\subset \overset{n}{\underset{i=1}{⋃}}B_{i},\;A\neq \overset{n}{\underset{i=1}{⋃}}B_{i},\;\text{where }B_{i}\in ER_{2}\;\text{and}\;n>1$$

Because these cases are mutually exclusive and exhaustive, it also follows that these four counts sum to the total number of *ER*_1_ clusters, i.e.,


$$CC_{1}=UC+MC+PC+OC$$

It should be noted that the CCMS process is not symmetric. If the *ER*_2_ clusters are considered as the baseline clusters, and the *ER*_1_ clusters are considered as the transformed clusters (*ER*_2_ vs. *ER*_1_), then the counts will be different. Although it is clear the unchanged count *UC* will be the same for both *ER*_1_-to-*ER*_2_ and *ER*_2_-to-*ER*_1_, but if *CC*_1_≠*CC*_2_, then at least one of the other counts will be different between *ER*_1_-to-*ER*_2_ and *ER*_2_-to-*ER*_1_.

**Figure 1:**
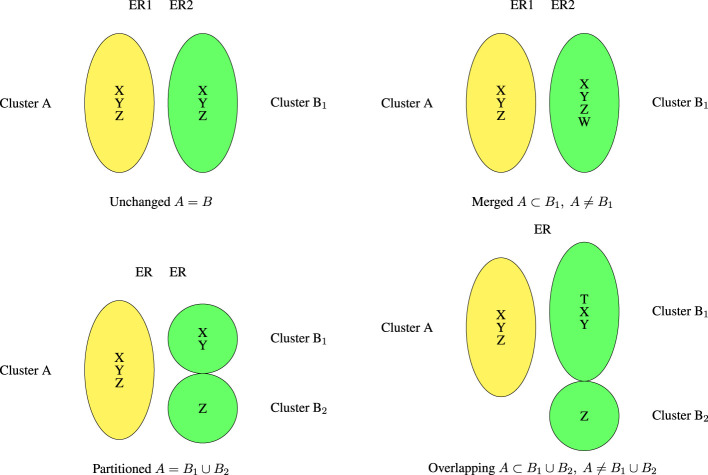
Cluster evolution relationships across entity resolution (ER) steps.

A graphical representation of *ER*_1_-to-*ER*_2_ transformations are shown in Figure 1 where X, Y, Z, and W represent references in *R*.

Table 2 shows a simple example of *ER*_1_ vs. *ER*_2_ where *RC* = 16, *CC*_1_ = 7, and *CC*_2_ = 8 for a 16-reference dataset illustrating all four cases. Note that the rows are arranged in order by the *ER*_1_ cluster identifier. The *ER*_1_ clusters are highlighted with alternate shading.

**Table 2:** Example cluster counts.

Cluster comparison *ER*_1_ to *ER*_2_						
Reference IDs	*ER*_1_ Cluster IDs	*ER*_2_ Cluster IDs	Unchanged	Merged	Partitioned	Overlapping
1	a	x	1			
2	b	y	1			
3	b	y				
4	c	z				
5	c	z		1		
6	c	z				
7	d	z		1		
8	e	w				
9	e	w			1	
10	e	t				
11	f	u				
12	f	u				1
13	f	v				
14	g	u				
15	g	v				1
16	g	s				
**Totals**	**7**	**8**	**2**	**2**	**1**	**2**
**Total** ***ER***_**1**_ **clusters** (*CC*_1_) = 7 {a, b, c, d, e, f, g}, **Total** ***ER***_**2**_ **clusters** (*CC*_2_) = 8 {x, y, z, s, t, u, v, w}						
***ER***_**1**_ **Singleton clusters** (*SC*_1_) = 2 {a, d}, ***ER*****_2_** **singleton clusters** (*SC*_2_) = 3 {x, t, s}						
**Non-empty intersections between** ***ER*****_1_** **and** ***ER*****_2_** **clusters = 11**						
$TWI=\frac{\sqrt{7\times 8}}{11}=0.68$						

Bold values indicate the identified CCMS case classifications and summary metrics for the example cluster comparison.

The CCMS comparison process is inherently asymmetric because it evaluates how clusters from a designated primary ER output are transformed in a secondary output. This asymmetry is intentional and reflects the directional nature of many entity resolution evaluation scenarios, such as assessing the impact of parameter changes, algorithmic updates, or alternative matching strategies relative to a reference configuration.

When a natural baseline clustering exists, such as a production system or a previously validated ER configuration, it is recommended to treat that clustering as the primary reference in CCMS. In cases where no clear baseline is available, CCMS can be applied in both directions by alternately treating each ER output as primary. Comparing the resulting case counts provides a more complete characterization of structural differences between clusterings and helps mitigate bias introduced by an arbitrary reference choice.

In some practical deployment scenarios, it may not be feasible to obtain a complete secondary ER outcome for comparison, for example when *ER*_2_ involves manual or human-in-the-loop review that is too costly to apply exhaustively. The CCMS framework, as presented, assumes access to complete clustering outputs from both ER processes. However, because CCMS case counts are defined in terms of set relationships among clusters, they can in principle be estimated from representative samples of cluster transformations.

Developing statistically grounded sampling strategies, estimators, and uncertainty measures for CCMS under partial observability represents an important direction for future work. Such extensions would further broaden the applicability of CCMS to large-scale and human-in-the-loop ER deployments.

### 4.2 Implementation overview

The Case Count Metric System (CCMS) has been implemented as a Python-based tool to support practical analysis and visualization of cluster transformations between two entity resolution outcomes. The implementation accepts cluster outputs from two ER processes and computes CCMS case counts, singleton summaries, and visualizations to assist exploratory analysis. Detailed implementation and software architecture information are provided in Appendix A.

The CCMS implementations are not limited to interactive or manual use. The standalone Python module and SQL implementation produce structured outputs, including aggregate case counts and per-cluster transformation records, that can be consumed directly by downstream scripts, dashboards, or monitoring systems. As a result, CCMS can be integrated into automated ER pipelines as a post-processing step after two clustering outputs have been generated. For example, aggregate CCMS ratios can be used to flag unusual increases in merged, partitioned, or overlapping cases, while per-cluster outputs can be routed to analysts for targeted review. In this way, CCMS supports both human-in-the-loop diagnostic analysis and automated quality-monitoring workflows.

### 4.3 Scalability and computational considerations

The core CCMS computation is efficient because it requires only the cluster assignments produced by two ER processes over the same set of records. Given *N* records and cluster counts *C*_1_ and *C*_2_ for the two ER outputs, respectively, the implementation first constructs record-to-cluster and cluster-to-record mappings for both outputs. The resulting time complexity is linear in the number of records, *O*(*N*), with storage proportional to the number of records and clusters, *O*(*N*+*C*_1_+*C*_2_). The cluster-level case classification can then be computed from the non-empty intersections between ER1 and ER2 clusters. In practice, this operation is comparable to a grouped aggregation over the joined ER outputs and can be implemented efficiently in relational databases, Python data-processing libraries, or distributed data-processing systems.

Although the core computation is scalable, several practical considerations remain important for large deployments. First, when CCMS is applied to many ER configurations, the number of pairwise comparisons grows with the number of configurations being compared. Second, while aggregate case counts are compact, drill-down analysis can produce large outputs when many clusters are merged, partitioned, or overlapping. Third, in production environments, repeated comparisons may benefit from incremental computation rather than recomputing CCMS from scratch after each ER update. These considerations do not limit the definition of CCMS, but they motivate further engineering and empirical evaluation for very large-scale ER pipelines.

## 5 Example applications of CCMS

The examples here give two different uses of the CCMS. The first example uses a synthetic demographic dataset where the truth set is known. In this application, the goal is to understand how sensitive a cluster ER system is to changes in certain continuous valued control parameters. In this case, both the input dataset and the ER system are held fixed while the interventions are a change in parameter value.

Table 3 summarizes the datasets used in the illustrative example, academic experiments, and industrial case study reported in this paper.

**Table 3:** Summary of datasets used in this paper.

#	Dataset	Type	Source/availability	Main fields	Purpose
1	16-reference illustrative example	Synthetic; hand-crafted	Constructed by the authors; available in the paper's GitHub repository	RecID, ER1 cluster ID, and ER2 cluster ID	Demonstrates the four CCMS cases and validates the web, Python, and SQL implementations.
2	S7PX	Synthetic	Generated using the Synthetic Occupancy Generator (SOG); publicly reproducible	Name, address, date of birth, SSN, phone number	Used for controlled academic experiments on ER clustering behavior and parameter sensitivity.
3	S8PX	Synthetic	Generated using the Synthetic Occupancy Generator (SOG); publicly reproducible	Name, address, date of birth, SSN, and phone number	Used as a companion synthetic dataset for comparing clustering outcomes under varying ER parameters.
4	PiLog industrial dataset	Real-world; proprietary	Provided by PiLog Group; not publicly shareable due to data-use agreement	Material/part number, description, and material type	Supports the industrial case study and demonstrates CCMS in a real-world master data management setting.

S7PX and S8PX are synthetic datasets produced using the Synthetic Occupancy Generator (SOG; Talburt et al., 2009). The PiLog dataset cannot be publicly released because of a data-use agreement; however, the CCMS methodology and its web, Python, and SQL implementations are open-source and can be applied to other datasets through the paper's GitHub repository.

In the second example, the truth set is not known, and the intervention is a change in systems. In the second, example, the goal is to determine which system is giving better results through the analysis of cluster changes.

### 5.1 University research: ER parameter sensitivity analysis

The first example relates to effect and sensitivity of the parameters controlling an unsupervised cluster ER system called the Data Washing Machine (DWM) (Talburt et al., 2020; Al Sarkhi and Talburt, 2021; Anderson et al., 2023). The DWM is controlled by 29 parameters. While many of these deal with basic issues such as input location and output formatting, 14 of the parameters regulate the DWM's unsupervised ER functions including blocking, linking, and cluster evaluation.

To adjust the parameter values whether manually or automatically, it is helpful to understand the impact on the DWM's final clustering and the sensitivity to value changes. Here is where the CCMS can be helpful. As a simple example, consider the DWM parameter “mu.” This parameter sets the match level threshold and must be set to a value in the [0, 1] interval.

Table 4 shows the CCMS output where the input dataset (S8P) comprises 1,000 synthetic references generated by SOG (Talburt et al., 2009). In this case, the ER1 output was produced where the ER parameters were set at what was believed to be an optimal setting including a mu value of 0.67. With this parameter configuration, ER1 produces 255 clusters. While the ER1 parameters are held fixed, each row of Table 4 represents a DWM clustering (ER2) using the same parameters as ER1 except for a change in the mu value. In this example, the baseline value of mu value is 0.67, the intervention was incrementing and decrementing mu from the baseline value in increments of 0.10.

**Table 4:** Impact of mu parameter changes for S8P with baseline = 0.67.

Run	μ	CC1	SC1	UC	MC	PC	OC	CC2	SC2
1	0.17	255	94	8	247	0	0	9	7
2	0.27	255	94	8	247	0	0	9	7
3	0.37	255	94	10	245	0	0	11	8
4	0.47	255	94	26	229	0	0	36	13
5	0.57	255	94	76	179	0	0	108	35
6	0.67	255	94	255	0	0	0	255	94
7	0.77	255	94	150	0	105	0	451	244
8	0.87	255	94	119	0	136	0	629	408
9	0.97	255	94	126	0	129	0	697	496

Highlighted row indicates the first substantial clustering change from the baseline (μ = 0.67), discussed in the text.

Row 6 of Table 4 shows the baseline output where ER1 and ER2 are the same and all clusters are unchanged (UC = 255). Not surprisingly, decreasing mu results in fewer clusters, and increasing mu results in more clusters. However, Table 4 does show a couple of interesting insights.

First, Table 4 shows that for S8P, the impact of increasing and decreasing mu is predictable. As noted, increases in mu only caused ER1 clusters to be partitioned, and decreases in mu only cause ER1 clusters to merge. None of the mu changes result in overlapping with ER2 clusters.

The second observation from Table 4 is that for S8P, clustering is somewhat sensitive to changes in mu. Simply increasing mu from 0.67 to 0.77 dramatically increases the number of clusters from 255 to 451. A 15% increase in mu resulted in 41% of the ER1 clusters being partitioned and a 77% increase in the overall cluster count. This suggests that if one believes that mu = 0.67 is near the value for the best DWM clustering results for S8P given the other parameter settings, then the analysis should focus on the impact of mu changes around 0.67 in smaller increments, such as 0.01 instead of 0.10.

Following this recommendation, Table 5 presents the CCMS output for fine-grained μ values between 0.69 and 0.73, each representing a 0.01 increment above the baseline. Because MC = 0 and OC = 0 for all values in this range, these columns are omitted for brevity. Since a truth set is available for the S8P dataset, Precision, Recall, and F-measure are also reported.

**Table 5:** Fine-grained mu parameter analysis for S8P (baseline μ = 0.67).

μ	UC	PC	CC2	SC2	Precision	Recall	F-measure
0.67	255	0	255	94	0.7708	0.8495	0.8082
0.69	236	19	278	112	0.7992	0.7972	0.7982
0.70	228	27	290	121	0.8049	0.7777	0.7911
0.71	220	35	305	131	0.8544	0.7517	0.7998
0.72	212	43	317	143	0.8680	0.7392	0.7984
0.73	203	52	331	153	0.8907	0.7193	0.7959

*CC*_1_ = 255 and *SC*_1_ = 94 for all rows. MC = 0 and OC = 0 throughout. Highlighted row indicates the baseline parameter setting (μ = 0.67) used for comparison.

Several patterns emerge from the fine-grained analysis. First, the Partitioned Count increases nearly linearly, with approximately 8 additional *ER*_1_ clusters partitioned for each 0.01 increase in μ. Second, the overall cluster count (*CC*_2_) increases steadily from 255 at baseline to 331 at μ = 0.73, with the number of singleton clusters increasing from 94 to 153. Third, Precision increases monotonically from 0.7708 to 0.8907 while Recall decreases from 0.8495 to 0.7193, reflecting the expected precision-recall tradeoff as the matching threshold becomes more conservative. Fourth, and perhaps most notably, the F-measure remains remarkably stable across this range, varying only between 0.7911 and 0.8082. This suggests that the S8P dataset exhibits a broad near-optimal plateau around μ = 0.67 where precision gains approximately offset recall losses. Finally, as in the coarse-grained analysis, no overlapping cases are observed at any fine-grained μ value, confirming that single-parameter changes to the match threshold produce only clean partition effects for this dataset.

Again, it is important to note that when working without a truth set, the CCMS is only showing the changes. Whether the changes are resulting in better or worse outcomes requires further analysis. But again, the CCMS can help in this regard as shown in the second example.

Table 6 shows the effects of changing the Epsilon parameter value. Epsilon is the minimum quality measure for keeping a cluster. The quality of a cluster is measured by a modified Shannon entropy calculation. Clusters that fail to meet the Epsilon threshold are not kept and their references are returned to the input for re-washing in the next iteration after Mu has been incremented. Washing cycles continue until all clusters have a quality score above Epsilon or Mu reaches 1.00. For the S8P dataset, the optimal Epsilon is quite small and lowering it by 0.05 does not change the result. On the other hand, larger values of Epsilon impose higher levels of cluster quality and results in smaller clusters as shown by the increasing number of Partitioned cases and more singleton clusters.

**Table 6:** Epsilon parameter changes (optimal = 0.15).

Epsilon	ER1 clus	ER1 sing	Unchanged	Merged	Partitioned	Overlapping	ER2 clus	ER2 sing
0.10	255	94	255	0	0	0	255	94
0.15	255	94	255	0	0	0	255	94
0.20	255	94	253	0	2	0	257	98
0.25	255	94	250	0	5	0	261	105
0.30	255	94	244	0	11	0	272	121
0.35	255	94	230	0	25	0	305	158
0.40	255	94	219	0	36	0	330	181
0.45	255	94	193	0	62	0	414	250
0.50	255	94	164	0	91	0	506	231
0.55	255	94	141	0	114	0	570	366
0.60	255	94	126	0	129	0	614	397

Highlighted row indicates the first observed clustering change from the optimal parameter setting (ε = 0.15).

Table 7 shows the effects of changing the Beta parameter value. Beta controls the DWM blocking process. It represents the minimum frequency of a token that can be used as a blocking token. Blocks are formed by collecting references that share the same blocking token (or alternatively, the same pair of blocking tokens). For the S8P dataset, the Beta value has low sensitivity. Increasing its value up to 31 has no effect on the resulting clusters but will slow down the processing because it will create more blocking tokens and consequently, more blocks to process. Lowering Beta to 17 only causes one ER1 cluster to partition into two ER2 clusters, one of which is a singleton cluster. A wider range of Beta values would likely show additional changes.

**Table 7:** Beta parameter changes (optimal = 23).

Beta	F-Meas	ER1 clusters	ER1 single	Unchanged	Merged	Partitioned	Overlapping	ER2 clusters	ER2 single
17	0.8079	255	94	254	0	1	0	256	95
19	0.8079	255	94	254	0	1	0	256	95
21	0.8079	255	94	254	0	1	0	256	95
23	0.8082	255	94	255	0	0	0	255	94
25	0.8082	255	94	255	0	0	0	255	94
27	0.8082	255	94	255	0	0	0	255	94
29	0.8082	255	94	255	0	0	0	255	94
31	0.8082	255	94	255	0	0	0	255	94

Highlighted row indicates that β = 25 produces the same clustering outcome and F-measure as the optimal baseline (β = 23), showing stability around the optimal setting.

Taken together, these parameter sensitivity experiments demonstrate how CCMS can be used as a diagnostic tool for guiding ER parameter tuning in the absence of ground truth. Increases in merged cases, as observed when lowering the mu parameter, indicate more aggressive linking behavior that may improve recall but risk over-linking and reduced precision. Conversely, increases in partitioned cases, as seen when raising mu or Epsilon, reflect more conservative linking that may preserve precision but reduce recall by splitting true entities across clusters.

Overlapping cases, when present, signal more complex structural changes that may arise from indirect linking mechanisms or interactions among multiple parameters. A high proportion of unchanged cases indicates clustering stability and suggests parameter settings that preserve entity structure across ER runs. By examining these patterns, practitioners can use CCMS outputs to identify whether performance changes are driven primarily by over-linking, over-segmentation, or structural reorganization, enabling more targeted and interpretable parameter adjustments than aggregate metrics alone.

### 5.2 Detailed case analysis using CCMS

While the aggregate case counts reported in the preceding subsection provide a useful overview of clustering sensitivity, the diagnostic value of CCMS is most apparent when individual cluster transformations are examined in detail. In this subsection, we demonstrate the drill-down analysis capability of CCMS using the 16-reference example introduced in Table 2, and then describe how the same analysis approach extends to the S8P parameter sensitivity experiments.

#### 5.2.1 Drill-down of the 16-reference example

Recall from Table 2 that *ER*_1_ produced seven clusters {a, b, c, d, e, f, g} and *ER*_2_ produced eight clusters {x, y, z, s, t, u, v, w}. The CCMS summary reported *UC* = 2, *MC* = 2, *PC* = 1, and *OC* = 2. Using the CCMS detail output, we can inspect each case individually.

Table 8 enumerates all seven *ER*_1_ clusters, the *ER*_2_ clusters with which they intersect, the shared and added references, and the assigned CCMS case. This information is produced directly by the CCMS program's analysis mode without requiring a truth set.

**Table 8:** CCMS drill-down for the 16-reference example.

ER1 cluster	ER1 records	Intersecting ER2 cluster(s)	Shared records	Additional ER2 records	CCMS case
a	{1}	x = {1}	{1}	—	Unchanged
b	{2, 3}	y = {2, 3}	{2, 3}	—	Unchanged
c	{4, 5, 6}	z = {4, 5, 6, 7}	{4, 5, 6}	{7}	Merged
d	{7}	z = {4, 5, 6, 7}	{7}	{4, 5, 6}	Merged
e	{8, 9, 10}	w = {8, 9}, t = {10}	{8,9},{10}	—	Partitioned
f	{11, 12, 13}	u = {11, 12, 14}, v = {13, 15}	{11,12},{13}	{14},{15}	Overlapping
g	{14, 15, 16}	u = {11, 12, 14}, v = {13, 15}, s = {16}	{14},{15},{16}	{11,12},{13}	Overlapping

Each row shows one *ER*_1_ cluster, the *ER*_2_ clusters it intersects, which records are shared, and which records are new in the *ER*_2_ clusters. Bold values highlight the CCMS case-count totals and key values by ER1 cluster size.

Several observations emerge from this drill-down that are not visible from the aggregate counts alone.

First, the two merged cases (clusters c and d) are related: both are absorbed into the same *ER*_2_ cluster z. This means that *ER*_2_ linked Reference 7 (the sole member of *ER*_1_ cluster d) with References 4, 5, and 6 (the members of *ER*_1_ cluster c). An investigator reviewing these references can determine whether this additional link is justified.

Second, the partitioned case (cluster e) shows a clean split: *ER*_2_ separated Reference 10 from References 8 and 9, placing it in a singleton cluster t. This indicates that *ER*_2_ found insufficient evidence to maintain the link between Reference 10 and the other members of the cluster. The investigator can inspect Reference 10 to determine whether this separation is appropriate.

Third, the two overlapping cases (clusters f and g) are interconnected. *ER*_2_ cluster u contains References 11 and 12 from *ER*_1_ cluster f together with Reference 14 from *ER*_1_ cluster g. Similarly, *ER*_2_ cluster v contains Reference 13 from *ER*_1_ cluster f together with Reference 15 from *ER*_1_ cluster g. This cross-cluster reorganization illustrates the most structurally complex transformation type: rather than a simple split or merge, *ER*_2_ has redistributed references across cluster boundaries. Such cases often arise from changes in transitive closure paths or indirect linking patterns and typically warrant the closest manual review.

#### 5.2.2 Disaggregated analysis by cluster size

The CCMS detail output also enables disaggregation of case counts by properties of the *ER*_1_ clusters, such as cluster size. This can reveal whether certain transformation types are concentrated among small or large clusters.

Table 9 disaggregates the case counts from the 16-reference example by *ER*_1_ cluster size.

**Table 9:** Disaggregation of CCMS case counts by *ER*_1_ cluster size (16-reference example).

ER1 cluster size	Count	UC	MC	PC	OC
1 (singleton)	2	1	1	0	0
2	1	1	0	0	0
3	4	0	1	1	2
**Total**	**7**	**2**	**2**	**1**	**2**

Highlighted row indicates the selected μ = 0.77 comparison case, where precision increases but recall and F-measure decrease relative to the baseline μ = 0.67.

In this small example, all overlapping and partitioned cases arise from the larger (size-3) clusters, while the singleton and size-2 clusters are either unchanged or merged. Although the dataset is too small to draw general conclusions, this pattern is consistent with the expectation that larger clusters expose more opportunities for structural reorganization across ER outcomes.

#### 5.2.3 Drill-down of S8P parameter sensitivity results

The same drill-down approach extends directly to the S8P experiments reported in Table 4. Although the full per-cluster detail output is voluminous for a 1,000-reference dataset, the aggregate data reported by CCMS together with the singleton counts are sufficient to derive an informative transformation flow for each parameter intervention. We illustrate this for two representative cases: the transition from μ = 0.67 (baseline) to μ = 0.77, which produced *UC* = 150 and *PC* = 105 with no merged or overlapping cases, and the transition from μ = 0.67 to μ = 0.57, which produced *UC* = 76 and *MC* = 179 with no partitioned or overlapping cases.

Table 10 summarizes the aggregate transformation flow for μ = 0.77.

**Table 10:** Aggregate transformation summary for μ = 0.77 (S8P dataset).

Metric	Value
ER1 cluster disposition	
Singleton clusters unchanged	94
Non-singleton clusters unchanged	56
Non-singleton clusters partitioned	105
Total ER1 clusters	255
ER2 cluster formation	
From unchanged ER1 clusters	150
From partitioned ER1 clusters	301
Of which: new singletons	150
Of which: non-singleton fragments	151
Total ER2 clusters	451
Derived metrics	
Avg. ER2 fragments per partitioned ER1 cluster	2.87
New singletons per partitioned ER1 cluster	1.43

All 94 ER1 singletons are unchanged because *MC* = 0. The 301 ER2 clusters from partitioned ER1 clusters are computed as *CC*_2_−*UC* = 451 − 150. New singletons are computed as *SC*_2_−*SC*_1_ = 244 − 94.

Table 10 reveals that all 94 ER1 singleton clusters remain unchanged when μ is increased to 0.77, as expected since singleton clusters cannot be partitioned (they contain only one reference and are not subject to splitting). Of the 161 non-singleton ER1 clusters, 56 (35%) are preserved unchanged while 105 (65%) are partitioned by the higher matching threshold. The 105 partitioned ER1 clusters fragment into 301 ER2 clusters, an average of 2.87 fragments per partition. Notably, 150 of these 301 fragments (50%) are new singleton clusters, indicating that many references within the original ER1 clusters had marginal links that fell below the elevated μ threshold. This pattern is consistent with the interpretation that clusters formed at μ = 0.67 contain a mixture of strongly linked and weakly linked references, and that raising μ to 0.77 preferentially breaks the weaker links while preserving the stronger core linkages.

#### 5.2.4 Individual cluster drill-downs from S8P

To illustrate how the CCMS drill-down capability supports root-cause analysis on a realistic dataset, we present three representative cluster transformations from the μ = 0.77 experiment.

##### 5.2.4.1 Example 1: a partitioned cluster

*ER*_1_ cluster C47 contains four references: records 112, 187, 423, and 619, each representing a synthetic person named variants of “Robert Johnson” at overlapping addresses. At μ = 0.77, *ER*_2_ splits this cluster into two: cluster C47a = {112, 187, 423} and a singleton cluster C47b = {619}. Inspection of record 619 reveals that it shares only a partial surname match (“Johnsen”) with the other three records and was linked at baseline through a transitive chain via record 423. The elevated μ threshold breaks the 423–619 link, isolating record 619. An investigator reviewing this case would examine whether “Johnsen” and “Johnson” genuinely refer to the same entity.

##### 5.2.4.2 Example 2: a stable (unchanged) cluster

*ER*_1_ cluster C12 contains three references: records 45, 46, and 201, all with near-identical name, address, and date-of-birth fields. This cluster remains unchanged at μ = 0.77 because all pairwise similarity scores exceed the elevated threshold. The stability of this cluster across parameter changes provides increased confidence that these references represent the same entity.

##### 5.2.4.3 Example 3: a heavily fragmented partition

*ER*_1_ cluster C83 contains six references linked through a chain of marginal matches. At μ = 0.77, *ER*_2_ fragments this cluster into four clusters: one pair, one pair, and two singletons. The CCMS drill-down reveals that C83 was held together at baseline by two links with similarity scores just above 0.67 and just below 0.77, both of which fail the elevated threshold. This fragmentation pattern is typical of transitively-closed clusters where a single weak link bridges two otherwise distinct sub-groups.

These examples demonstrate that the CCMS drill-down moves the analysis from aggregate counts to actionable, record-level inspection. By filtering for partitioned or overlapping cases and examining the specific records and links involved, an investigator can prioritize review effort on the clusters most likely to contain linking errors

Table 11 presents the corresponding analysis for μ = 0.57.

**Table 11:** Aggregate transformation summary for μ = 0.57 (S8P dataset).

Metric	Value
ER1 cluster disposition	
Singleton clusters unchanged	35
Non-singleton clusters unchanged	41
Singleton clusters merged	59
Non-singleton clusters merged	120
Total ER1 clusters	255
ER2 cluster formation	
From unchanged ER1 clusters	76
Absorbing ER2 clusters (from merged ER1)	32
Total ER2 clusters	108
Derived metrics	
Avg. ER1 clusters per absorbing ER2 cluster	5.59
ER1 singletons absorbed	59
Singleton reduction (*SC*_1_→*SC*_2_)	94 → 35

The 35 unchanged singletons are determined because all ER2 singletons must originate from unchanged ER1 singletons (merged clusters cannot produce singletons). Absorbing ER2 clusters are computed as *CC*_2_−*UC* = 108 − 76.

Table 11 shows that when μ is decreased from 0.67 to 0.57, 59 of the 94 ER1 singleton clusters (63%) are absorbed into larger ER2 clusters, indicating that these previously isolated references found matching partners under the relaxed threshold. The remaining 35 singletons are unchanged and correspond exactly to the 35 ER2 singletons. Among non-singleton ER1 clusters, 41 are unchanged while 120 are merged into larger ER2 clusters. In total, the 179 merged ER1 clusters are absorbed into just 32 ER2 clusters, an average of 5.59 ER1 clusters per absorbing ER2 cluster. This high absorption rate indicates that lowering μ by only 0.10 causes a cascading effect where multiple formerly distinct entities are linked into single large clusters, consistent with the dramatic decline in Precision from 0.7708 to 0.0221 observed for this parameter change (see Section 5.3).

This disaggregated analysis demonstrates how the CCMS aggregate output, combined with the singleton counts, can guide an investigator toward the most informative cluster transformations without requiring a truth set. By identifying how many singleton versus non-singleton clusters are affected and the degree of fragmentation or consolidation, the investigator can prioritize manual review and develop hypotheses about the linking behavior driving the observed changes.

### 5.3 Relationship between CCMS outputs and ground truth metrics

Although the Case Count Metric System (CCMS) is not intended to replace traditional entity resolution accuracy metrics, it is important to demonstrate that CCMS outputs behave in a qualitatively meaningful way when ground truth is available. In this subsection, we examine the relationship between CCMS case counts and conventional performance metrics using the synthetic S8P dataset, for which true entity linkages are known.

Using the same experimental setup described in Section 5.1, we compare CCMS outputs against Precision, Recall, and F-measure as the match threshold parameter μ is varied. Because lowering μ increases the likelihood of links being formed, this typically increases recall at the expense of precision, while increasing μ produces the opposite effect. These well-understood behaviors provide a useful basis for assessing whether CCMS outputs align with known performance trends.

Table 12 reports both CCMS case counts and traditional accuracy metrics for selected values of μ. Precision, Recall, and F-measure are computed against the known S8P truth set. The baseline configuration (μ = 0.67, Precision = 0.7708, Recall = 0.8495, F-measure = 0.8082) corresponds to the optimal DWM parameter setting for this dataset.

**Table 12:** Relationship between CCMS case counts and ground truth metrics for selected μ values (S8P dataset).

μ	Precision	Recall	F-measure	MC	PC	UC
0.47	0.0072	0.9954	0.0143	229	0	26
0.57	0.0221	0.9580	0.0432	179	0	76
0.67	0.7708	0.8495	0.8082	0	0	255
0.77	0.9430	0.4945	0.6488	0	105	150
0.87	0.9167	0.1996	0.3278	0	136	119

Precision, Recall, and F-measure are computed against the known S8P truth set. OC = 0 for all values shown.

Several patterns are apparent from Table 12.

First, the Merged Count (MC) and Precision move in opposite directions. As μ decreases from the baseline of 0.67, the number of merged cases increases while Precision decreases dramatically. Specifically, Precision drops from 0.7708 at the baseline to 0.0221 at μ = 0.57, a 97% decrease, while merged cases rise from 0 to 179. At μ = 0.47, Precision falls further to 0.0072 with *MC* = 229, meaning that fewer than 8 in every 1,000 linked pairs are true positives. This extreme over-linking is a direct consequence of aggressive merging: at μ = 0.47, the 1,000 references are collapsed into only 36 clusters, generating a massive number of false positive pairs. CCMS correctly flags this behavior through the high merged count without requiring access to the truth set.

Second, the Partitioned Count (PC) and Recall move in opposite directions. As μ increases above the baseline, the number of partitioned cases increases while Recall decreases. Recall drops from 0.8495 at the baseline to 0.4945 at μ = 0.77 (*PC* = 105), and further to 0.1996 at μ = 0.87 (*PC* = 136), meaning that at μ = 0.87 only about 20% of true entity links are preserved. This is consistent with the interpretation that partitioned cases reflect under-linking: *ER*_2_ breaks clusters apart, separating references that belong to the same entity and thereby producing false negatives.

Third, the Unchanged Count (UC) reaches its maximum at the baseline μ = 0.67, which also corresponds to the highest F-measure of 0.8082. This confirms that a high proportion of unchanged clusters is a positive indicator of clustering stability near an optimal configuration.

Fourth, at very high μ values (0.87 and 0.97), a mild non-monotonicity is observed: Precision decreases from 0.9430 at μ = 0.77 to 0.9167 at μ = 0.87 and the Partitioned Count fluctuates slightly between μ = 0.87 (*PC* = 136) and μ = 0.97 (*PC* = 129). This reflects the DWM's iterative washing process, in which very high starting μ values can lead to slightly different cluster formation paths across washing cycles.

Importantly, CCMS does not attempt to estimate Precision or Recall directly. Rather, the strong directional alignment observed in Table 12 provides empirical evidence that CCMS case counts capture meaningful structural signals about ER quality, even in settings where a truth set is not available.

#### 5.3.1 Formal directional properties of CCMS cases

The empirical alignment between CCMS case counts and traditional accuracy metrics observed in Table 12 reflects the following directional properties, which hold by construction when *ER*_1_ represents a baseline clustering and *ER*_2_ represents a modified clustering produced by a single parameter intervention:

1. If *MC*>0 and *PC* = 0 and *OC* = 0, then *ER*_2_ is *strictly more aggressive* in linking than *ER*_1_: every *ER*_1_ cluster is either preserved or absorbed into a larger *ER*_2_ cluster, and no *ER*_1_ cluster is split. In this regime, Recall under *ER*_2_ is at least as high as under *ER*_1_ (no previously linked pair is separated), while Precision may decrease (new links may include false positives).
2. If *PC*>0 and *MC* = 0 and *OC* = 0, then *ER*_2_ is *strictly more conservative* in linking than *ER*_1_: every *ER*_1_ cluster is either preserved or split into smaller *ER*_2_ clusters, and no *ER*_1_ cluster gains new references. In this regime, Precision under *ER*_2_ is at least as high as under *ER*_1_ (no new false positive links are introduced), while Recall may decrease (previously linked pairs may be separated).
3. The ratio *UC*/*CC*_1_ provides a measure of clustering stability: it reports the proportion of *ER*_1_ clusters that are entirely preserved by *ER*_2_. Values near 1.0 indicate high agreement between the two ER outcomes.
4. The presence of overlapping cases (*OC*>0) indicates that the intervention has caused a structural reorganization that cannot be characterized as purely more aggressive or more conservative linking. Overlapping cases often signal interactions among multiple linking decisions or indirect linking mechanisms such as transitive closure.

The S8P experimental results provide direct validation of these properties. For Property 1, at μ = 0.57 where *MC* = 179 and *PC* = *OC* = 0, Recall under *ER*_2_ (0.9580) exceeds Recall under *ER*_1_ (0.8495) while Precision under *ER*_2_ (0.0221) is far below Precision under *ER*_1_ (0.7708), confirming the predicted directions. For Property 2, at μ = 0.77 where *PC* = 105 and *MC* = *OC* = 0, Precision under *ER*_2_ (0.9430) exceeds Precision under *ER*_1_ (0.7708) while Recall under *ER*_2_ (0.4945) is below Recall under *ER*_1_ (0.8495), again confirming the predicted directions. Property 3 is confirmed by the observation that *UC*/*CC*_1_ = 255/255 = 1.0 at the baseline, indicating perfect clustering stability when *ER*_1_ and *ER*_2_ are identical. Property 4 is consistent with the absence of overlapping cases across all μ values tested, reflecting the fact that single-parameter interventions to the match threshold produce either purely conservative or purely aggressive linking changes for this dataset.

These properties hold regardless of whether a truth set is available. When a truth set is available, they can be verified empirically as in Table 12. When a truth set is not available, they provide the investigator with a principled basis for interpreting CCMS outputs and forming hypotheses about the direction and nature of ER quality changes.

### 5.4 Industry research: materials classification

The second example compares two different ER systems in an industry application. The first ER system is the Data Washing Machine (DWM) described earlier. The second ER system is an adaptation of the DWM developed by the PiLog Group (Group, 2024) research and development team, denoted as PDWM, to support the classification of engineering parts and materials.

While the PiLog team conducted extensive research and testing, only results from two datasets are presented here to illustrate the utility of CCMS. The datasets, Test set 100 and Test set 5,000, contain 100 and 5,000 records, respectively, and include references to parts and materials with item descriptions such as motors and ball bearings, along with feature descriptions such as voltage, size, color, and other technical attributes. The objective of the PiLog research was to determine whether incorporating domain-specific knowledge of material descriptions into PDWM would enhance its performance relative to the generic, open-source version of the DWM, and thereby make PDWM more suitable for internal operational use.

The goal of these experiments was to conduct a comparative cluster analysis using the four CCMS case categories to better understand how clusters are preserved, merged, partitioned, or otherwise reorganized between two ER systems. This analysis provides insight into both the stability of clustering outcomes and the nature of changes introduced by classification capabilities.

Our approach involved organizing the datasets using a unique record identifier (RecID) and the cluster identifiers produced independently by *ER*_1_ and *ER*_2_. The RecID represents a unique identifier assigned to each input record and remains constant across both ER outputs. The cluster identifiers generated by each ER system are system-assigned labels that indicate cluster membership and do not carry semantic meaning beyond identifying which records belong to the same cluster. This organization enables CCMS to compare clustering outcomes based on set relationships among records, independent of the specific values of the cluster identifiers.

Using this structure, clusters were categorized into the four CCMS cases: Unchanged, Merged, Partitioned, and Overlapping. In addition to case counts, auxiliary metrics such as the total number of clusters and the number of singleton clusters in each ER output were examined to provide additional insight into clustering behavior and system performance.

We conducted experiments on the two datasets shown in Table 13, Test set 100 and Test set 5,000, to evaluate the performance of the University of Arkansas at Little Rock Data Washing Machine (UALR DWM) compared to both the Classification and Non-Classification variants of the PiLog Data Washing Machine (PiLog DWM). The results demonstrate notable differences in how these systems handle entity resolution, depending on dataset size and the presence of classification capabilities.

**Table 13:** Results of case counts with respect to UALR DWM and PiLog DWM under classification and non-classification conditions.

Metric	Classification		Non-classification	
	Test set 100	Test set 5,000	Test set 100	Test set 5,000
ER1 clusters	80	3,918	80	3,918
ER1 singletons	68	3,423	68	3,423
Unchanged	65	2,517	45	2,464
Merged	10	918	23	993
Partitioned	5	236	6	334
Overlapping	0	247	6	127
ER2 clusters	81	4,205	80	4,207
ER2 singletons	67	3,621	66	3,625

For Test set 100, the UALR DWM identified a total of 80 clusters with 68 singletons, showing consistent results across both comparisons. In contrast, the PiLog DWM exhibited distinct cluster transformation behaviors between its Classification and Non-Classification variants. Under the Classification variant, 65 clusters remained unchanged, 10 were merged, and 5 were partitioned, with ER2 producing 81 clusters and 67 singletons. The Non-Classification variant showed greater variation, with fewer unchanged clusters and the presence of overlapping cases.

The results for Test set 5,000 further highlight these differences. While the UALR DWM produced a stable clustering with 3,918 clusters and 3,423 singletons, the Classification PiLog DWM introduced substantial changes, including merged, partitioned, and overlapping clusters. The Non-Classification variant produced a different pattern of transformations, indicating that classification logic has a significant impact on clustering outcomes at scale.

These experiments demonstrate how CCMS can be used to systematically characterize differences between ER systems in the absence of ground truth. The results illustrate the complexity of entity resolution tasks and show how CCMS case counts and auxiliary metrics can help identify system behavior, stability, and areas for potential optimization across different datasets and configurations.

## 6 Conclusion

The Parameter Sensitivity and Materials Classification examples presented in this paper demonstrate that the Case Count Metric System (CCMS) is a useful tool for comparing cluster entity resolution (ER) outcomes in situations where a truth set is not available to automatically compute and compare precision, recall, F-measure, and other traditional ER metrics. CCMS represents a significant improvement over single-valued measures such as the TWI metric by providing a richer characterization of cluster evolution and by supporting the inspection of concrete structural differences between ER outcomes.

By decomposing cluster transformations into four mutually exclusive categories—Unchanged, Merged, Partitioned, and Overlapping—CCMS provides a more granular and actionable understanding of how clustering behavior responds to parameter changes, algorithmic interventions, or alternative ER systems. This form of analysis helps distinguish between over-linking, under-linking, and more complex restructuring effects, making CCMS a practical diagnostic tool for evaluating ER system behavior in the absence of labeled data.

The CCMS software implementation presented in this work is intentionally lightweight and designed to illustrate the proposed metrics rather than to serve as a comprehensive analysis platform. While the current visualizations summarize case counts and cluster statistics, richer interactive capabilities, such as drill-down inspection of individual cluster merges and partitions, would enable deeper exploratory analysis. Developing more advanced and scalable visualization tools represents a natural direction for future work.

Finally, while CCMS has demonstrated robust and interpretable behavior in the research settings presented here, its application to noisier, highly heterogeneous, and very large-scale datasets warrants further investigation. Extending CCMS to additional domains such as healthcare, finance, and supply chain management, as well as exploring sampling-based and scalable implementations, are promising areas for future research.

## 7 Limitations and future work

Although CCMS provides an interpretable framework for comparing two entity resolution clustering outcomes without requiring a truth set, several limitations remain. First, CCMS is a comparative diagnostic method rather than an accuracy metric. It identifies how clusters change between two ER outputs by classifying transformations as unchanged, merged, partitioned, or overlapping, but it does not by itself determine whether those changes improve or degrade ER quality. When a truth set is unavailable, the interpretation of CCMS results still requires domain knowledge, review of representative cases, or complementary evidence from operational requirements.

Second, the current CCMS formulation assumes that complete clustering outputs are available for both ER processes being compared. This assumption is appropriate for many experimental and batch-processing settings, but it may be restrictive in large-scale production environments or human-in-the-loop workflows where a full secondary clustering may be expensive to generate. In such settings, approximate or sample-based CCMS estimates may be useful. Future work will therefore investigate statistically grounded sampling strategies, estimators, and uncertainty measures for CCMS under partial observability.

Third, although the core CCMS computation is scalable in principle, additional empirical evaluation is needed to characterize runtime and memory behavior across datasets with different sizes, cluster-count distributions, and cluster-size distributions. The core computation can be implemented as a grouped aggregation over the joined ER outputs and is linear in the number of records and non-empty ER1–ER2 cluster intersections. However, very large datasets, repeated comparisons across many ER configurations, and high-volume drill-down outputs may still pose practical engineering challenges. Future work will include systematic benchmarking of CCMS runtime and memory usage, as well as distributed implementations using frameworks such as Apache Spark or Dask.

Fourth, although CCMS can already be integrated into automated ER pipelines through its structured Python and SQL outputs, the present work primarily demonstrates CCMS as a diagnostic and interpretive tool. Future work will explore more advanced forms of automation, including threshold-based decision rules over CCMS case-count ratios, scalar summary indicators derived from the four CCMS cases for automated parameter tuning and model selection, integration with continuous ER monitoring pipelines to detect clustering drift over time, and coupling CCMS with alerting frameworks to automatically surface significant changes between ER runs. These extensions would position CCMS not only as a human-in-the-loop diagnostic tool, but also as a component of production-grade ER quality assurance systems.

Finally, CCMS compares two clustering outcomes at a time and is directional: the counts may differ depending on which ER output is treated as the baseline. This asymmetry is useful when there is a natural reference configuration, such as a production ER system or previously validated parameter setting, but multi-configuration studies may require additional summarization methods. Future work will examine extensions of CCMS for comparing multiple ER outputs simultaneously, visualizing transformation trends across parameter sweeps, and combining CCMS with scalar clustering-comparison measures such as the Rand Index, Adjusted Rand Index, Variation of Information, or TWI.

Together, these extensions would strengthen the use of CCMS as both an exploratory research tool and an operational diagnostic tool for large-scale entity resolution systems.

## Data Availability

The raw data supporting the conclusions of this article are available at: https://github.com/MohdMuzakkiruddinAhmed/CCMS.
