# Supplementary material for: Case count metric for comparative analysis of entity resolution results
Source: Front Big Data. 2026 Jun 11;9:1736939. doi: 10.3389/fdata.2026.1736939 (PMC13293780; doi:10.3389/fdata.2026.1736939)
Supplement: Supplementary file 1 [file Supplementary_file_1.pdf]

A IMPLEMENTATION OF THE CASE COUNT METRIC SYSTEM

This appendix provides implementation details for the Case Count Metric System (CCMS). Three complementary implementations are described: a web-based application for interactive analysis (Section A.1), a standalone Python module for programmatic use and reproducibility (Section A.2), and a set of SQL queries for database-resident ER outputs (Section A.3).

The complete source code, example datasets, and usage instructions are publicly available at `https://github.com/MohdMuzakkiruddinAhmed/CCMS`.

A.1 Web Application Implementation

The Case Count Metric System (CCMS) has been implemented as a Python-based web application using Flask. The implementation enables users to analyze, compare, and visualize the outcomes of Entity Resolution (ER) clustering processes when a truth set is unavailable. This section describes the design and functionality of the implementation.

A.1.1 Design and Architecture

The application framework is developed using Flask to manage server-side operations such as file uploads, cluster analysis, and result rendering, as shown in Figure 1. It is integrated with a responsive HTML interface built using Bootstrap for enhanced usability and DataTables for efficient display of input data.

The system accepts two CSV input files containing the fields *RecID*, *ER1 ClusterID*, and *ER2 ClusterID* from two Entity Resolution (ER) systems. The output consists of a detailed cluster transformation analysis that includes textual summaries of cluster cases (Unchanged, Merged, Partitioned, and Overlapping), visualizations such as bar and pie charts illustrating the distribution of these cases, and identification of singleton clusters in both ER_1 and ER_2.

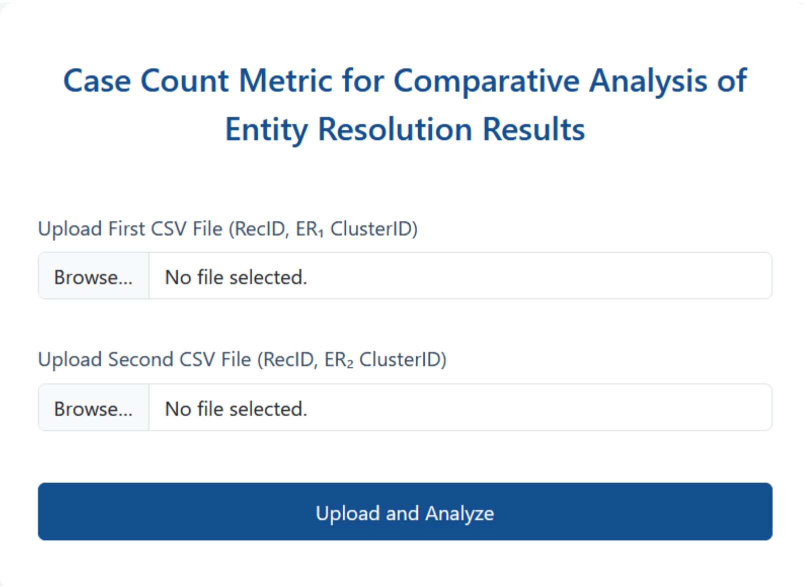

Figure 1. User Input Interface for the Case Count Metric System. The interface allows users to upload two CSV files corresponding to ER_1 and ER_2 cluster outputs and supports comparative entity resolution analysis through visualization and metric computation.

A.1.2 Key Functional Components

Cluster Analysis and Classification

Clusters from ER_1 and ER_2 are analyzed to classify transformations into four distinct cases:

- **Unchanged:** Clusters that are identical in both ER_1 and ER_2.
- **Merged:** Clusters from ER_1 that are subsets of larger clusters in ER_2.
- **Partitioned:** Clusters from ER_1 that are split into multiple smaller clusters in ER_2.
- **Overlapping:** Clusters from ER_1 that overlap with multiple clusters in ER_2, forming complex relationships.

A.1.3 Example Logic for Case Determination

The following Python code illustrates the logical conditions used to identify *identical* and *partitioned* cluster cases within the web application:

```
def is_identical(cluster):
    return len(cluster['er2_clusters']) == 1 and \
           cluster['size_er1'] == len(cluster['er2_references'])

def is_partitioned(cluster):
    return len(cluster['er2_clusters']) > 1 and \
           cluster['size_er1'] == len(cluster['er2_references'])
```

A.1.4 Visualizations

ECharts is utilized to generate interactive bar and pie charts that illustrate the outcomes of cluster transformations. These visualizations provide a comparative overview of:

- Case count distributions across transformation types.
- Proportions of total clusters between ER_1 and ER_2.

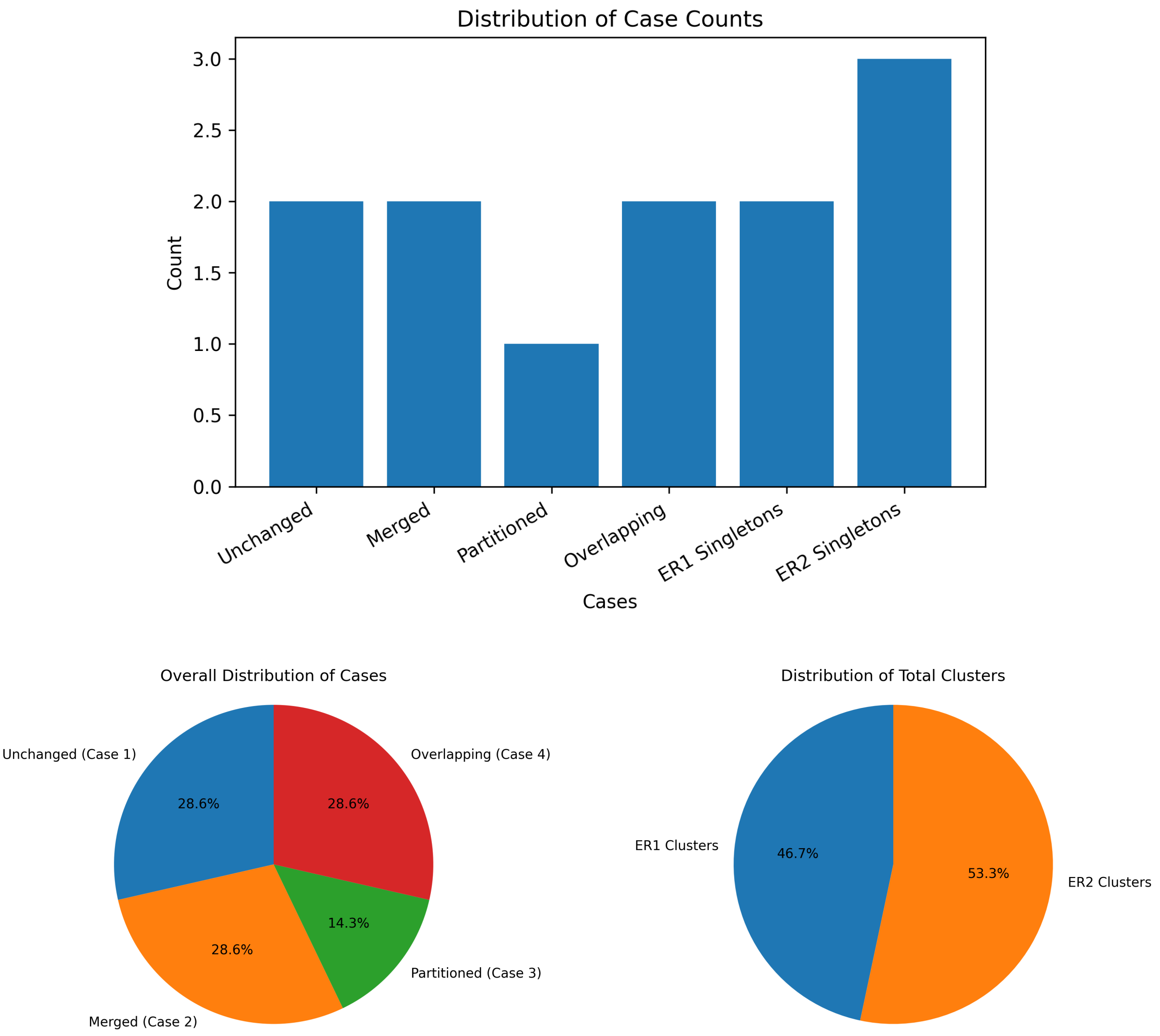

Figure 2. Comparative Visualizations of Case Counts and Cluster Proportions (Illustrative Example). Top: bar chart of CCMS case counts. Middle: pie charts showing case proportions. Bottom: ER_1 vs. ER_2 cluster totals. This example uses $RC = 16$ references, with $CC1 = 7$ clusters in ER_1 and $CC2 = 8$ clusters in ER_2.

A.1.5 Singleton Detection

Singleton detection is implemented to identify isolated clusters in both ER_1 and ER_2. The following function provides an example implementation:

Listing 1. Example Python function for singleton cluster detection

```
def determine_singletons(df, clusters):
    er1_singletons = {
        cluster: refs for cluster, refs in clusters.items()
        if len(refs['er2_references']) == 1
```

```
    }
    return er1_singletons
```

A.1.6 Summary Report Generation

The summary report consolidates computed metrics and provides a textual overview of the clustering outcomes between ER_1 and ER_2. An example output from the web application is shown below:

```
Detailed Summary Report
ER1 as primary and ER2 as secondary:
Unchanged (Case 1): 2
Merged (Case 2): 2
Partitioned (Case 3): 1
Overlapping (Case 4): 2
ER1 clusters: 7

ER2 as primary and ER1 as secondary:
Unchanged (Case 5): 2
Merged (Case 6): 3
Partitioned (Case 7): 1
Overlapping (Case 8): 2
ER2 clusters: 8

Total clusters: 15

Singletons:
ER1 Singletons: 2
ER2 Singletons: 3
```

A.1.7 Example Use Case

Input:

- `RecID, ER1 ClusterID`
- `RecID, ER2 ClusterID`

Output:

- Visualizations showing the distribution of Unchanged, Merged, Partitioned, and Overlapping cluster cases.
- A detailed textual report summarizing case counts, singleton clusters, and overall cluster transformations.

The web-based implementation of CCMS provides a practical and user-friendly platform for interactive analysis of cluster differences between two entity resolution processes. The complete source code for the web application is available at `https://github.com/MohdMuzakkiruddinAhmed/CCMS/tree/claude/setup-ccms-repo-e1ajb/webapp`.

A.2 Standalone Python Implementation

In addition to the web application described above, a standalone Python module has been developed to support programmatic use, batch processing, and integration into automated analysis workflows. This implementation requires only the Python standard library and produces both aggregate case counts and a per-cluster detail list that supports the drill-down analyses demonstrated in Section 5.

The `compute_ccms` function accepts two dictionaries mapping record identifiers to cluster identifiers and returns a dictionary containing all CCMS counts and detail records. The module includes functions for loading CSV input files, printing aggregate CCMS reports, and printing drill-down details filtered by case type (Unchanged, Merged, Partitioned, or Overlapping). It can be used as an importable library or invoked directly from the command line with two CSV files as arguments.

The complete standalone Python implementation, including the core `compute_ccms` module, CSV loading utilities, report generation functions, and usage instructions, is publicly available at `https://github.com/MohdMuzakkiruddinAhmed/CCMS/tree/claude/setup-ccms-repo-e1ajb/ccms`. The core algorithm is implemented in `https://github.com/MohdMuzakkiruddinAhmed/CCMS/blob/claude/setup-ccms-repo-e1ajb/ccms/core.py`.

A.2.1 Example Output for the 16-Reference Dataset

Applying the standalone implementation to the 16-reference dataset from Table **??** produces the following output, which corresponds exactly to the drill-down analysis presented in Table **??**:

```
ER1 clusters (CC1): 7  Singletons (SC1): 2
ER2 clusters (CC2): 8  Singletons (SC2): 3
Unchanged (UC):   2
Merged    (MC):   2
Partitioned (PC): 1
Overlapping (OC): 2
Total (UC+MC+PC+OC): 7

=== MERGED CASES ===

--- ER1 Cluster: c [Merged] ---
  ER1 records: [4, 5, 6]
  -> ER2 cluster z: shared [4, 5, 6]

--- ER1 Cluster: d [Merged] ---
  ER1 records: [7]
  -> ER2 cluster z: shared [7]

=== PARTITIONED CASES ===

--- ER1 Cluster: e [Partitioned] ---
  ER1 records: [8, 9, 10]
  -> ER2 cluster w: shared [8, 9]
  -> ER2 cluster t: shared [10]
```

```
=== OVERLAPPING CASES ===

--- ER1 Cluster: f [Overlapping] ---
  ER1 records: [11, 12, 13]
  -> ER2 cluster u: shared [11, 12]
  -> ER2 cluster v: shared [13]

--- ER1 Cluster: g [Overlapping] ---
  ER1 records: [14, 15, 16]
  -> ER2 cluster u: shared [14]
  -> ER2 cluster v: shared [15]
  -> ER2 cluster s: shared [16]
```

This confirms that the standalone implementation reproduces the expected case classifications and supports the same drill-down analysis available through the web application.

A.3 SQL Implementation

When ER outputs are stored in a relational database, the CCMS computation can be expressed in standard SQL without requiring data export to flat files. The queries below assume a single table `records` with columns `RecID`, `ER1_ClusterID`, and `ER2_ClusterID`. This implementation is functionally equivalent to both the web application and the standalone Python module, and produces identical case counts and drill-down details.

Listing 2. SQL queries for CCMS computation.

```sql
-- Step 1: For each ER1 cluster, compute the number
-- of distinct intersecting ER2 clusters and the
-- total number of records in their union.

CREATE VIEW er1_analysis AS
SELECT
    a.ER1_ClusterID,
    COUNT(DISTINCT a.RecID) AS er1_size,
    COUNT(DISTINCT a.ER2_ClusterID)
        AS er2_cluster_count,
    (SELECT COUNT(DISTINCT r2.RecID)
     FROM records r2
     WHERE r2.ER2_ClusterID IN (
         SELECT DISTINCT ER2_ClusterID
         FROM records
         WHERE ER1_ClusterID = a.ER1_ClusterID
     )
    ) AS er2_union_size
FROM records a
GROUP BY a.ER1_ClusterID;
```

```sql
-- Step 2: Classify each ER1 cluster into a
-- CCMS case.

CREATE VIEW er1_cases AS
SELECT
    ER1_ClusterID,
    er1_size,
    er2_cluster_count,
    er2_union_size,
    CASE
        WHEN er2_cluster_count = 1
         AND er1_size = er2_union_size
            THEN 'Unchanged'
        WHEN er2_cluster_count = 1
         AND er1_size < er2_union_size
            THEN 'Merged'
        WHEN er2_cluster_count > 1
         AND er1_size = er2_union_size
            THEN 'Partitioned'
        WHEN er2_cluster_count > 1
         AND er1_size < er2_union_size
            THEN 'Overlapping'
    END AS case_type
FROM er1_analysis;

-- Step 3: Aggregate case counts.

SELECT case_type, COUNT(*) AS case_count
FROM er1_cases
GROUP BY case_type
ORDER BY case_type;

-- Step 4 (optional): Drill-down for a specific
-- case type, e.g., Overlapping.

SELECT
    c.ER1_ClusterID,
    c.er1_size,
    r.ER2_ClusterID,
    COUNT(r.RecID) AS shared_records
FROM er1_cases c
JOIN records r
    ON r.ER1_ClusterID = c.ER1_ClusterID
WHERE c.case_type = 'Overlapping'
GROUP BY c.ER1_ClusterID, c.er1_size,
```

```
         r.ER2_ClusterID
ORDER BY c.ER1_ClusterID, r.ER2_ClusterID;
```

The SQL implementation is particularly suitable for large-scale deployments where ER outputs are maintained in database systems. Step 4 demonstrates how the drill-down capability of CCMS can be realized directly in SQL by filtering on a specific case type and reporting the intersecting ER2 clusters for each affected ER1 cluster. Analogous queries can be constructed for Merged and Partitioned cases by changing the `WHERE` clause filter.
